# Treatment outcomes and survival in patients with primary central nervous system lymphomas treated between 1995 and 2010 – a single centre report

**DOI:** 10.2478/v10019-012-0048-5

**Published:** 2012-11-09

**Authors:** Barbara Jezersek Novakovic

**Affiliations:** Department of Medical Oncology, Institute of Oncology Ljubljana, Ljubljana, Slovenia

**Keywords:** primary central nervous system lymphomas, treatment outcomes, survival

## Abstract

**Background.:**

Primary central nervous system lymphomas (PCNSL) are rare variants of extranodal non-Hodgkin’s lymphomas that are nowadays primarily treated with high-dose methotrexate or methotrexate-based chemotherapy with or without radiation therapy. The optimal treatment of PCNSL is still unknown and there are differences in clinical practice.

**Patients and methods.:**

With a retrospective research we evaluated our series of patients with PCNSL in regards to the patient’s characteristics, treatment results, disease specific survival and overall survival. Fifty nine patients who attended the Institute of Oncology Ljubljana between 1995 and 2010 were treated according to the protocol that was valid at the time of the patient’s admission. Between 1995 and 1999, the systemic treatment was classical CHOP (cyclophosphamide, doxorubicin, vincristine, steroids) chemotherapy, and later on high-dose methotrexate either alone or in combination with other agents. From 1999 onwards, radiation therapy was applied according to the patient’s age and response to chemotherapy, prior to that all patients treated with CHOP were also irradiated. Patients ineligible for the systemic treatment were treated with sole radiation therapy.

**Results.:**

There was a strong female predominance in our series and the median age at diagnosis was 59.8 years. Patients had predominantly aggressive B cell lymphomas (69.5%), one patient had marginal cell lymphoma and two patients T cell lymphoma. In total, 20.3% of patients were treated just with chemotherapy, 33.9% with combined therapy and 42.4% with sole radiation therapy. The overall response rate to the primary treatment in patients treated with sole chemotherapy was 33.3%, in patients treated with combined therapy 65% and in patients treated only with radiation therapy 56%, respectively. In terms of response duration, significantly better results were achieved with combined therapy or radiation therapy alone compared to sole chemotherapy (p<0.0006). The median overall survival of the whole cohort was 11 months and the overall survival was significantly affected by the patient’s age. The longest overall survival was observed in patients treated with combined therapy (median survival of 39 months). Patients treated just with radiation therapy had a median overall survival of 9 months and those treated with sole chemotherapy of 4.5 months, respectively.

**Conclusions.:**

The treatment outcomes in ordinary clinical practice are definitely inferior to the ones reported in clinical trials. The now standard treatment with high-dose methotrexate with or without radiation therapy is sometimes too aggressive and, therefore, a careful selection on the basis of patient’s age, performance status and concomitant diseases of those eligible for such treatment is mandatory. According to our results from a retrospective study, radiation therapy should not be excluded from the primary treatment.

## Introduction

Primary central nervous system lymphomas (PCNSL) are quite rare variants of extranodal non-Hodgkin’s lymphomas (NHL) that involve the brain, eyes, leptomeninges or spinal cord without evidence of systemic disease.^1^,^2^ The largest part of cases of non-AIDS related PCNSL are diagnosed in patients between 45 and 70 years of age.^3^,^4^ The incidence increases with advancing age and just a few cases have been reported in children where more frequently other tumors of the central nervous system are observed.^5^,^6^ Men and women are reported to be equally affected.

The most important risk factor for the development of PCNSL is immunodeficiency (*e.g*. HIV infection, iatrogenic immune suppression, congenital immune deficiency) and this may play a role in the pathogenesis of disease.^7^,^8^ Five distinct clinicopathological entities have been described – intracranial lesion (solitary or multiple); diffuse leptomeningeal or periventricular lesions; vitreous/uveal deposits; intradural spinal cord lesion and nerve seeking lymphoma (neurolymphomatosis).^4^,^5^,^9^

Presenting symptoms and signs of the disease vary, depending on the site of the involvement and they may include focal neurological deficits, neuropsychiatric symptoms, signs of raised intracranial pressure, seizures, and ocular symptoms – as they appear in other primary or secondary brain tumors.^10^–^12^

Untreated PCNSL have a rapidly fatal course, with a survival of approximately 1.5 months from the time of diagnosis. Survival after the whole brain radiation therapy ranges from 10 to 18 months, but was reported to rise to an average of 44 months following chemotherapy and radiation therapy, or chemotherapy alone.^13^–^19^ Yet, radiation therapy is associated with a high incidence of neurotoxicity, which is however not seen after the radiation therapy of patients with brain metastases of solid tumors because of their shorter survival or lower tumor dose.^20^ Although currently available therapeutic regimes prolong the survival, they are in contrast to therapies used to treat systemic lymphomas,^21^,^22^ not curative in most patients.

The optimal treatment of PCNSL is unknown and there are differences in clinical practice. The PCNSL are primarily treated with a high-dose methotrexate or methotrexate-based chemotherapy with or without radiation therapy. The role of radiation therapy is controversial due to its late toxicity, especially in older adults .^17^–^19^, ^23^–^27^ Treatment decisions should, therefore, take into consideration both response rates and impact on the quality of life.

With this retrospective research we evaluated our series of patients with PCNSL in regards to the patient’s characteristics, treatment results, disease specific survival and overall survival.

## Patients and methods

Fifty nine patients with PCNSL who attended the Institute of Oncology Ljubljana between 1995 and 2010 were identified from the database of the Cancer Registry of Slovenia. Patients who had any evidence of systemic disease (*i.e*. 10 patients from the primary database) were excluded from further evaluation. Patients with PCNSL were treated according to the protocol that was valid at the time of the patient’s admission. Between 1995 and 1999, patients were treated with classical CHOP (cyclophosphamide, doxorubicin, vincristine, steroids) chemotherapy, intrathecal applications of methotrexate and cytarabine and radiation therapy (radiation to the whole brain with 30–36 Gy and 10 to 14 Gy boost on primary tumor localization). Those ineligible for systemic therapy were treated with radiation therapy only. From 1999 onwards, patients were treated with a high-dose methotrexate (3 to 5 g/m^2^) either alone or in combination with a high-dose cytarabine (2 to 3 g/m^2^ twice daily for two consecutive days) or other blood-brain barrier passing agents (vincristine, procarbazine, carmustine). Radiation therapy was applied according to the patient’s age and response to chemotherapy. The patients’ characteristics, patohistological diagnosis, disease stage, response to treatment and survival data were taken from patients’ records. The treatment response was re-evaluated according to the International Primary CNS Lymphoma Collaborative Group Guidelines for Response Assessment^28^ and the disease-free and overall survivals were assessed by means of Kaplan Meier survival curves. For the determination of statistical differences the log rank test and Chi-square test were applied.

## Results

### Patients’ characteristics and treatment

The patients’ characteristics are given in Table 1. None of the patients was HIV positive. Patients had predominantly aggressive B cell lymphomas (69.5%), one patient had marginal cell lymphoma and two patients T cell lymphoma (3.4%). Majority of patients (93.2%) had intracranial lesions and in twenty (33.2%) these lesions were multiple. Deep brain structures were affected in 54.2% of patients. The leptomeningeal involvement was confirmed in 18.6% of the patients, cerebrospinal fluid cytology was positive in 10.2% of patients. Spinal cord lesions were detected in 6.8% of patients, while vitreous/uveal deposits and nerve seeking lymphoma have not been observed in our series.

Performance status prior to the treatment was poor, 71.1% of patients had the performance status of 2 or more. Thirteen patients also suffered from serious concomitant diseases. The international prognostic index (IPI) fell in the high intermediate or in the high risk group in 40.7% of patients. The prognostic factors reported by the International Extranodal Lymphoma study group were not followed systematically because the data on elevated cerebrospinal fluid (CSF) protein concentration were frequently not precise in patients’ records.

Details on the primary treatment are given in Table 2. Just five patients had no surgery, while all the others had at least stereotactic biopsy to diagnose lymphoma. Chemotherapy was given to 54.2% of patients, slightly more than half of them received a high-dose methotrexate either as a single agent (two patients) or in combination with a high-dose cytarabine (nine patients), CHOP (one patient) or with other blood-brain barrier passing agents (five patients). Fifteen patients received other regimens – eight patients from the beginning of our series (*i.e*. prior to 1999) were treated with CHOP and seven patients with either middle-dose (500 mg/m^2^) methotrexate (five patients) or just corticosteroids (two patients). Patients received median 4 cycles of CHOP chemotherapy (range 2 to 6), median 3 cycles of high-dose methotrexate (range 1 to 6) and median 3 cycles of high-dose methotrexate when combined with high-dose cytarabine (range 1 to 4). Twenty patients were treated after chemotherapy also with radiation therapy (one in a palliative setting) and in twenty-five patients radiation therapy was the only primary treatment. Two patients were treated just with radical surgery.

### Treatment outcomes

**FIGURE 1:**
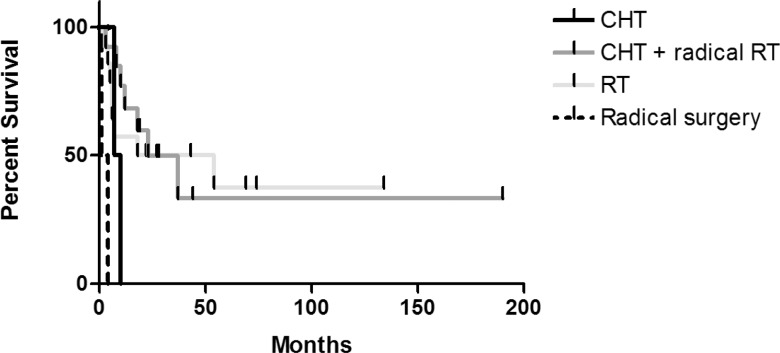
The disease-free survival for different treatment modalities.

The overall response rate to the primary treatment in patients treated with sole chemotherapy was 33.3%, in patients treated with chemotherapy followed by radiation therapy 65% and in patients treated only with radiation therapy 56%, respectively. In total, nineteen patients (57.6%) relapsed from the achieved complete or partial response. In fifteen patients, the relapse occurred in the central nervous system (CNS) (one of them had a concomitant systemic relapse) while five patients relapsed just outside the CNS. The disease-free survival for different treatment modalities is given in Figure 1. In terms of response duration, significantly better results were achieved with the combined therapy or radiation therapy alone compared to sole chemotherapy or radical surgery (p<0.0006).

**FIGURE 2:**
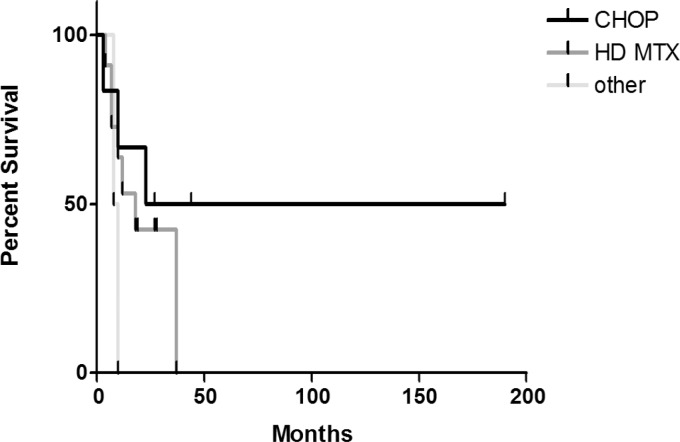
The disease-free survival after different chemotherapeutic regimens.

When taking in account different chemotherapy regimens, the best outcomes were achieved with the combination of a high-dose methotrexate and a high-dose cytarabine since the overall response rate was 55.5%. In this group, three patients achieved complete remission and two patients partial remission all of them except one receiving a full course of treatment (4 cycles). In three patients, stable disease was observed after 2 cycles and another patient progressed after the first cycle. However, the disease-free survival was better in case of CHOP regimen (which was in all cases followed with radiation therapy) compared to regimens comprising a high-dose methotrexate, yet insignificantly (p=0.29) (Figure 2).

The median overall survival of the whole cohort was 11 months (95% CI: 0.71 – 21.29) The overall survival was significantly affected by patient’s age – the median overall survival in patients aged below 60 years was namely 27 months (95% CI: 0.00 – 71.96) while in patients aged over 60 years it was just 7 months (95% CI: 2.81 – 11.19) (p=0.006). Six patients (10%) survived more than 105 months after the diagnosis of PCNSL has been made – all of them were aged below 60 years. Altogether, these patients had the diffuse large B cell histology and were treated as follows: three patients with radical radiotherapy, one with a high-dose methotrexate and radiation therapy, one with CHOP chemotherapy and radiation therapy and one with dexamethasone and radiation therapy.

**FIGURE 3:**
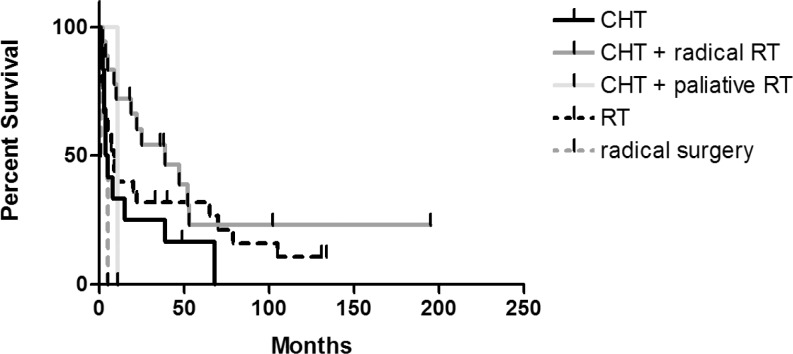
The overall survival for different treatment modalities.

The overall survival for different treatment modalities is presented in Figure 3. The longest overall survival was observed in patients treated with the combined treatment (median survival of 39 months, 95% CI: 10.41 – 67.59). This group comprised eight patients treated systemically with CHOP regimen, nine patients treated with a high-dose methotrexate (either as a single agent or in combination with other agents) and two patients treated with corticosteroids. Patients treated just with radiation therapy had a median overall survival of 9 months (95% CI: 4.14 – 13.87) and those treated with sole chemotherapy of 4.5 months (95% CI: 1.74 – 6.26), respectively. The group of patients treated with sole chemotherapy comprised eight patients treated with a high-dose methotrexate (either as a single agent or in combination with other agents) and four patients treated with a middle-dose methotrexate in combination with other blood-brain barrier passing agents. Just four of these patients achieved the complete remission with the primary systemic treatment while the others either progressed in the course of the systemic therapy (five patients) or died of treatment complications (three patients). The differences between the curves were not statistically significant (p=0.069). All three toxic deaths occurred in episodes of febrile neutropenia in patients suffering from severe mucositis following a high-dose methotrexate. Surprisingly, no toxic deaths were observed in the group of patients receiving a high-dose methotrexate in combination with a high-dose cytarabine.

**FIGURE 4:**
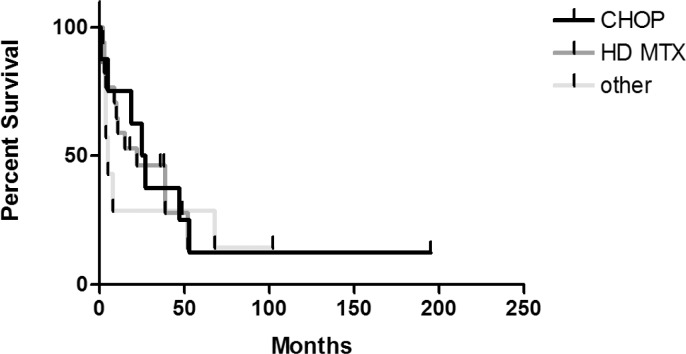
The overall survival after different chemotherapeutic regimens.

As for different chemotherapeutic regimens, again the longest median overall survival was achieved with the CHOP regimen (median 26 months, 95% CI: 13.91 – 36.09). Yet, we have to take in account that all these patients were also treated with radiation therapy. The differences between the survival curves were statistically insignificant (p=0.93) (Figure 4). On the other hand, the overall survival of the subgroup of patients treated with a high-dose methotrexate and a high-dose cytarabine was longer (yet insignificantly) compared to other patients treated with a high-dose methotrexate (33 months, 95% CI: 12.01 – 53.99, versus 22 months, 95% CI: 0.30 – 43.70).

**FIGURE 5:**
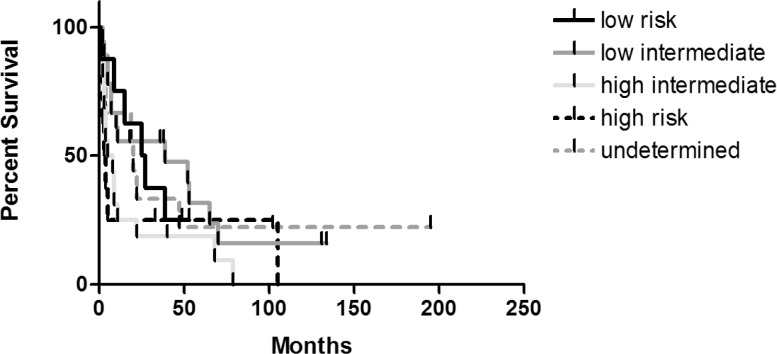
Overall survival according to different IPI categories.

Finally, we also determined the overall survival of patients in different international prognostic index (IPI) categories (Figure 5). The median overall survival of the low risk group was 26 months (95% CI: 8.37 – 41.63) and 39 months (95% CI: 0.00 – 101.98) in the low intermediate risk group, respectively. As expected, the median overall survival was shorter in the high intermediate risk group (6.5 months, 95% CI: 0.00 – 12.84) and in the high risk group (3.5 months, 95% CI: 0.23 – 5.77). The differences between the curves were insignificant (p=0.27).

## Discussion

In the article, the PCNSL patients treated at the Institute of Oncology Ljubljana between 1995 and 2010 were analysed in regards to the patient’s characteristics, treatment results, disease specific survival and overall survival.

Our series included almost twice as many females as males. This is different from the reports of Villano *et al.*^2^ and Uhm *et al*.^29^ who reported male predominance in their series. Only Lim *et al.*^30^ reported female predominance but in PCNSL other than the diffuse large B cell lymphoma. The median age at diagnosis of PCNSL in immunocompetent patients was reported to be 55 years^31^ which is slightly lower compared to the median age of 59.8 years in our patients. The diagnosis of PCNSL was made by the histological examination of a stereotactic biopsy in 22 patients and by the partial or radical resection in 32 patients. In 5 patients, the diagnosis was due to their poor performance status established by the cytological examination of the cerebrospinal fluid. Patients had predominantly aggressive B cell lymphomas (69.5%), among which there were 39 cases (66%) of the diffuse large B cell lymphoma, one case of immunoblastic lymphoma and one case of Burkitt’s lymphoma. One patient had marginal cell lymphoma (1.7%) and two patients T cell lymphoma (3.4%). In fifteen patients we diagnosed unspecified B cell lymphomas. The percentage of aggressive lymphomas is lower than the one reported by Miller *et al*. (89%)^3^, most probably on account of a large proportion of determined unspecified B cell lymphomas. We believe that with a more adequate surgical sampling and if the patients had not received corticosteroids prior to the procedure, the percentage of unspecified B cell lymphomas would have been lower, most probably on account of a higher proportion of the aggressive B cell lymphomas.

Multiple lesions were detected in 33.2% of patients which is substantially lower than 66% reported by Uhm *et al*.^29^ Also the involvement of deep brain structures (54.2%) was in our series observed more rarely than in the study of Uhm *et al.*^29^ where they found it in 78% of patients. Leptomeningeal involvement was identified in 18.6% of the patients and cerebrospinal fluid cytology was positive in 10.2% of patients while Uhm *et al.*^29^ reported of 38% of patients with the diffuse large B cell PCNSL having positive cerebrospinal fluid cytology. The majority of our patients showed poor performance status since 67.7% had the performance status of 3 and 4. The series of Lim *et al.*^30^ and Uhm *et al.*^29^ on the other hand included just 13% and 38% of patients with performance status of 3 and 4, respectively. Moreover, 22% of our patients suffered from serious concomitant diseases.

Two patients were, because of their poor performance status, treated just with radical surgery, they survived one and five months from the diagnosis of PCNSL, respectively. Prior to 1999, all patients eligible for the systemic treatment were treated with CHOP chemotherapy and intrathecal applications of chemotherapy, which was in all cases combined with radical radiotherapy. Surprisingly, in these patients the overall response rate to chemotherapy was 25%, however, the complete response was achieved in six patients (75%) following primary chemotherapy and radiotherapy. The median overall survival in this group was 26 months which was longer than in patients treated with a high-dose methotrexate (22 months) but shorter than in the subgroup of patients treated with a high-dose methotrexate in combination with a high-dose cytarabine (33 months). There was also one long term survivor (more than 105 months) in this group. These observations are discordant with the observations of O’Neill *et al.*, Schultz *et al*., and Shibamoto *et al*., who obtained unsatisfactory results with the anthracyclines and cyclophosphamide in combination with radiotherapy.^23^,^24^,^32^ On the other hand, treatment results with a high-dose methotrexate (alone or in combinations) in our series were inferior to the ones reported in the literature. Namely, the overall response rate of 55.5% in case of combination of a high-dose methotrexate and a high-dose cytarabine was lower than the one reported for a high-dose methotrexate in mono-therapy by Glass *et al*.^33^ and other authors^14^,^34^ and substantially lower than the 69% overall response rate reported by Ferreri *et al.* with the same combination in a randomized trial.^35^ Also, the survival in the group receiving a high-dose methotrexate regimens (46.3% at two years) was inferior to the two year survival of 60–65% reported by the same authors.^14^,^33^,^34^ We can only speculate about the underlying causes for such discrepant results between CHOP and high-dose methotrexate treatments. First of all, all patients treated with CHOP were also treated with radical radiotherapy while in the group of patients treated with a high-dose methotrexate only nine (52.9%) were irradiated. There were also three treatment related deaths in the group of patients who received methotrexate suggesting that this kind of treatment might possibly be too aggressive for some of them. Hypothetically, the fundamental shift in the biology of the PCNSLs between 1958 and 1989 described by Miller *et al.*^3^ could be another explanation since our patients were treated with CHOP just until 1999.

Most consistent results in our series were obtained with radiation therapy – namely, the overall response rate was 56% in patients treated with sole radiotherapy and 65% in those treated with combined therapy. Also the median disease-free survival in our group of patients treated just with radiation therapy was longer compared to the data of Nelson *et al*.^36^ and Laack *et al.*^37^ who reported that the disease recurred in more than 90% of patients within one year of treatment. The median overall survival of this group, however, was just 9 months compared to a median survival time of 23 or 6 to 8 months for those less than or greater than 60 years of age, respectively, reported by the same authors.^36^,^37^ Still, the five year overall survival was 32% which is higher than 3%–26% reported by Nelson *et al*.^36^ and Laperriere *et al*.^38^ Three out of six long term survivors were treated just with the radical radiation therapy. The median disease-free survival of patients treated with chemotherapy combined with the radiation therapy was 23 months and the median overall survival 39 months, respectively. This strategy produced a five year survival of 23.2% falling in the range of 22%–40% reported by other authors.^13^,^39^,^40^ Again, three out of six long term survivors were treated with the combined therapy.

The overall survival in our series was significantly affected by the patient’s age – it was namely substantially shorter in patients aged over 60 years. This is in agreement with other reports of treatment results of central nervous system tumors.^10^,^13^,^23^,^24^,^41^ On the other hand, the IPI that had been established as a good predictor of the survival in patients with systemic aggressive lymphomas, however, in case of PCNSL did not prove especially informative. Possibly this goes at least partially on account of a large proportion of patients in whom the IPI remained undetermined.

In conclusion, the treatment outcomes in ordinary clinical practice are definitely inferior to the ones reported in clinical trials. The now standard treatment with a high-dose methotrexate or methotrexate-based chemotherapy with or without radiation therapy is sometimes too aggressive and, therefore, a careful selection on the basis of the patient’s age, performance status and concomitant diseases of those eligible for such treatment is mandatory. According to our results, the radiation therapy should not be excluded from the primary treatment. These recommendations are, however, based on a retrospective study.

## Figures and Tables

**TABLE 1:** Characteristics of 59 patients with primary central nervous system lymphomas

**Gender**	**Males 20; Females 39**	**F/M ratio: 1.95**

**Age at diagnosis**	**14–85 years**	**Median: 59.8 years**
**Histology**		
Diffuse large B cell lymphoma (DLBCL)	39	66.1%
Immunoblastic lymphoma	1	1.7%
Burkitt’s lymphoma	1	1.7%
Marginal cell lymphoma	1	1.7%
Unspecified B cell lymphoma	15	25.4%
T cell lymphoma	2	3.4%
**Site of disease**		
Cerebral hemisphere	20	33.9%
Deep structures	32	54.2%
Cerebellum	3	5.1%
Spinal cord	4	6.8%
**Multiple lesions**		
Yes	20	33.9%
**Positive cerebrospinal fluid cytology**		
Yes	6	10.2%
No	51	86.4%
Unknown	2	3.4%
**Leptomeningeal involvement (MRI or positive CSF cytology)**		
Yes	11	18.6%
No	47	79.7%
Unknown	1	1.7%
**Performance status (ECOG) prior to treatment**		
0	3	5.1%
1	13	22.0%
2	2	3.4%
3	10	16.9%
4	30	50.8%
Unknown	1	1.7%
**Serious concomitant diseases**		
Yes	13	22.0%
**IPI**		
0	3	5.1%
1	5	8.5%
2	18	30.5%
3	16	27.1%
4	8	13.6%
Undetermined	9	15.3%

MRI = magnetic resonance imaging; CSF = cerebrospinal fluid; ECOG = Eastern Cooperative Oncology Group; IPI = international prognostic index

**TABLE 2:** Primary treatment of primary central nervous system lymphomas

**Surgery**		
None	5	3.4%
Biopsy	22	37.3%
Radical	19	32.2%
Non radical	13	22.0%
**Chemotherapy (CHT)**		
None	27	45.8%
HD MTX	17	28.8%
Other	15	25.4%
**Radiation therapy (RT)**		
None	14	23.7%
Radical	37	62.7%
Non radical	8	13.6%
**Primary treatment**		
CHT	12	20.3%
CHT + radical RT	19	32.2%
CHT + palliative RT	1	1.7%
RT	25	42.4%
Radical surgery	2	3.4%

HD MTX – high dose methotrexate

## References

[1] Hoffman S, Propp JM, McCarthy BJ (2006). Temporal trends in incidence of primary brain tumors in the United States, 1985–1999. Neuro Oncol.

[2] Villano JL, Koshy M, Shaikh H, Dolecek TA, McCarthy BJ (2011). Age, gender, and racial differences in incidence and survival in primary CNS lymphoma. Br J Cancer.

[3] Miller DC, Hochberg FH, Harris NL, Gruber ML, Louis DN, Cohen H (1994). Pathology with clinical correlations of primary central nervous system non-Hodgkin’s lymphoma. The Massachusetts General Hospital experience 1958–1989. Cancer.

[4] Fine HA, Mayer RJ (1993). Primary central nervous system lymphoma. Ann Intern Med.

[5] Hochberg FH, Miller DC (1988). Primary central nervous system lymphoma. J Neurosurg.

[6] Kachanov DY, Dobrenkov KV, Shamanskaya TV, Abdullaev RT, Savkova RF (2008). Solid tumors in young children in Moscow Region of Russian Federation. Radiol Oncol.

[7] Schabet M (1999). Epidemiology of primary CNS lymphoma. J Neurooncol.

[8] Bhagavathi S, Wilson JD (2008). Primary central nervous system lymphoma. Arch Pathol Lab Med.

[9] Herrlinger U, Schabet M, Clemens M, Kortmann RD, Petersen D, Will BE (1998). Clinical presentation and therapeutic outcome in 26 patients with primary CNS lymphoma. Acta Neurol Scand.

[10] Bataille B, Delwail V, Menet E, Vandermarcq P, Ingrand P, Wager M (2000). Primary intracerebral malignant lymphoma: report of 248 cases. J Neurosurg.

[11] Linnert M, Iversen HK, Gehl J (2012). Multiple brain metastases – current management and perspectives for treatment with electrochemotherapy. Radiol Oncol.

[12] Tezcan Y, Koc M (2011). 3-D conformal radiotherapy with concomitant and adjuvant temozolomide for patients with glioblastoma multiforme and evaluation of prognostic factors. Radiol Oncol.

[13] Abrey LE, DeAngelis LM, Yahalom J (1998). Long-term survival in primary CNS lymphoma. J Clin Oncol.

[14] Cher L, Glass J, Harsh GR, Hochberg FH (1996). Therapy of primary CNS lymphoma with methotrexate-based chemotherapy and deferred radiation therapy: preliminary results. Neurology.

[15] Dahlborg SA, Petrillo A, Crossen JR, Roman-Goldstein S, Doolittle ND, Fuller KH (1998). The potential for complete and durable response in nonglial primary brain tumors in children and young adults with enhanced chemotherapy delivery. Cancer J Sci Am.

[16] Schlegel U, Pels H, Glasmacher A, Kleinschmidt R, Schmidt-Wolf I, Helmstaedter C (2001). Combined systemic and intraventricular chemotherapy in primary CNS lymphoma: a pilot study. J Neurol Neurosurg Psychiatry.

[17] Blay JY (1997). Primary cerebral non-Hodgkin lymphoma in non-immunocompromised subjects. Bull Cancer.

[18] Pech IV, Peterson K, Cairncross JG (1998). Chemotherapy for brain tumors. Oncology (Williston Park).

[19] Ferreri AJ, Reni M, Villa E (2000). Therapeutic management of primary central nervous system lymphoma: lessons from prospective trials. Ann Oncol.

[20] Stanic K, Kovac V (2010). Prophylactic cranial irradiation in patients with small-cell lung cancer: the experience at the Institute of Oncology Ljubljana. Radiol Oncol.

[21] Horvat M, Jezersek Novakovic B (2010). Effect of response quality and line of treatment with rituximab on overall and disease-free survival of patients with B-cell lymphoma. Radiol Oncol.

[22] Gregoric B, Zadnik V, Jezersek Novakovic B (2012). The diffuse large B-cell lymphoma – where do we stand now in everyday clinical practice. Radiol Oncol.

[23] O’Neill BP, O’Fallon JR, Earle JD, Colgan JP, Brown LD, Krigel RL (1995). Primary central nervous system non-Hodgkin’s lymphoma: survival advantages with combined initial therapy. Int J Radiat Oncol Biol Phys.

[24] Schultz C, Scott C, Sherman W, Donahue B, Fields J, Murray K (1996). Preirradiation chemotherapy with cyclophosphamide, doxorubicin, vincristine, and dexamethasone for primary CNS lymphomas: initial report of radiation therapy oncology group protocol 88-06. J Clin Oncol.

[25] Ferreri AJ, Reni M, Pasini F, Calderoni A, Tirelli U, Pivnik A (2002). A multi-center study of treatment of primary CNS lymphoma. Neurology.

[26] Shibamoto Y, Ogino H, Suzuki G, Takemoto M, Araki N, Isobe K (2008). Primary central nervous system lymphoma in Japan: changes in clinical features, treatment, and prognosis during 1985–2004. Neuro Oncol.

[27] Joerger M, Huitema AD, Krähenbühl S, Schellens JH, Cerny T, Reni M (2010). Methotrexate area under the curve is an important outcome predictor in patients with primary CNS lymphoma: A pharmacokinetic-pharmacodynamic analysis from the IELSG no. 20 trial. Br J Cancer.

[28] Abrey LE, Batchelor TT, Ferreri AJ, Gospodarowicz MJ, Pulczynski EJ, Zucca E (2005). Report of an international workshop to standardize baseline evaluation and response criteria for primary CNS lymphoma. J Clin Oncol.

[29] Uhm JE, Kim KH, Yi SY, Chang MH, Park KW, Kong DS (2009). A retrospective study to compare two methotrexate-based ragimens for primary central nervous system lymphoma. Leuk Lymphoma.

[30] Lim T, Kim SJ, Kim K, Lee Ji, Lim do H, Lee DJ (2011). Primary CNS lymphoma other than DLBCL: a descriptive analysis of clinical features and treatment outcomes. Ann Hematol.

[31] Herrlinger U, Schabet M, Bitzer M, Petersen D, Krauseneck P (1999). Primary central nervous system lymphoma: from clinical presentation to diagnosis. J Neurooncol.

[32] Shibamoto Y, Sasai K, Oya N, Hiraoka M (1999). Systemic chemotherapy with vincristine, cyclophosphamide, doxorubicin and prednisolone following radiotherapy for primary central nervous system lymphoma: a phase II study. J Neurooncol.

[33] Glass J, Gruber ML, Cher L, Hochberg FH (1994). Preirradiation methotrexate chemotherapy of primary central nervous system lymphoma: Long-term outcome. J Neurosurg.

[34] O’Brien PC, Roos DE, Liew KH, Trotter GE, Barton MB, Walker QJ (1996). Preliminary results of combined chemotherapy and radiotherapy for non-AIDS primary central nervous system lymphoma. Trans-Tasman Radiation Oncology Group. Med J Aust.

[35] Ferreri AJ, Reni M, Foppoli M, Martelli M, Pangalis GA, Frezzato M (2009). High-dose cytarabine plus high-dose methotrexate versus high-dose methotrexate alone in patients with primary CNS lymphoma: a randomised phase 2 trial. Lancet.

[36] Nelson DF, Martz KL, Bonner H, Nelson JS, Newall J, Kerman HD (1992). Non-Hodgkin’s lymphoma of the brain: can high-dose, large volume radiation therapy improve survival? Report on a prospective trial by the Radiation Therapy Oncology Group (RTOG): RTOG 8315. Int J Radiat Oncol Biol Phys.

[37] Laack NN, Ballman KV, Brown PB, O’Neill BP, North Central Cancer Treatment Group (2006). Whole-brain radiotherapy and high-dose methylprednisolone for elderly patients with primary central nervous system lymphoma: Results of North Central Cancer Treatment Group (NCCTG) 96-73-51. Int J Radiat Oncol Biol Phys.

[38] Laperriere NJ, Cerezo L, Milosevic MF, Wong CS, Patterson B, Panzarella T (1997). Primary lymphoma of brain: results of management of a modern cohort with radiation therapy. Radiother Oncol.

[39] Brada M, Hjiyiannakis D, Hines F, Traish D, Ashley S (1998). Short intensive primary chemotherapy and radiotherapy in sporadic primary CNS lymphoma (PCL). Int J Radiat Oncol Biol Phys.

[40] O’Brien P, Roos D, Pratt G, Liew K, Barton M, Poulsen M (2000). Phase II multicenter study of brief single-agent methotrexate followed by irradiation in primary CNS lymphoma. J Clin Oncol.

[41] Niemiec M, Głogowski M, Tyc-Szczepaniak D, Wierzchowski M, Kępka L (2011). Characteristics of long-term survivors of brain metastases from lung cancer. Rep Pract Oncol Radiother.

